# Convergence of miR-143 overexpression, oxidative stress and cell death in HCT116 human colon cancer cells

**DOI:** 10.1371/journal.pone.0191607

**Published:** 2018-01-23

**Authors:** Sofia E. Gomes, Diane M. Pereira, Catarina Roma-Rodrigues, Alexandra R. Fernandes, Pedro M. Borralho, Cecília M. P. Rodrigues

**Affiliations:** 1 Research Institute for Medicines (iMed.ULisboa), Faculty of Pharmacy, Universidade de Lisboa, Lisbon, Portugal; 2 UCIBIO, Departamento de Ciências da Vida, Faculty of Sciences and Technology, New University of Lisbon, Caparica, Portugal; University of South Alabama Mitchell Cancer Institute, UNITED STATES

## Abstract

MicroRNAs (miRNAs) regulate a wide variety of biological processes, including tumourigenesis. Altered miRNA expression is associated with deregulation of signalling pathways, which in turn cause abnormal cell growth and de-differentiation, contributing to cancer. miR-143 and miR-145 are anti-tumourigenic and influence the sensitivity of tumour cells to chemotherapy and targeted therapy. Comparative proteomic analysis was performed in HCT116 human colon cancer cells stably transduced with miR-143 or miR-145. Immunoblotting analysis validated the proteomic data in stable and transient miRNA overexpression conditions in human colon cancer cells. We show that approximately 100 proteins are differentially expressed in HCT116 human colon cancer cells stably transduced with miR-143 or miR-145 compared to Empty control cells. Further, Gene Ontology and pathway enrichment analysis indicated that proteins involved in specific cell signalling pathways such as cell death, response to oxidative stress, and protein folding might be modulated by these miRNAs. In particular, antioxidant enzyme superoxide dismutase 1 (SOD1) was downregulated by stable expression of either miR-143 or miR-145. Further, SOD1 gain-of-function experiments rescued cells from miR-143-induced oxidative stress. Moreover, miR-143 overexpression increased oxaliplatin-induced apoptosis associated with reactive oxygen species generation, which was abrogated by genetic and pharmacological inhibition of oxidative stress. Overall, miR-143 might circumvent resistance of colon cancer cells to oxaliplatin via increased oxidative stress in HCT116 human colon cancer cells.

## Background

MicroRNAs (miRNAs) are small non-coding RNAs that regulate gene expression in a post-transcriptional manner, by inhibiting protein translation, and mRNA deadenylation and decay [1, 2]. miRNAs undergo several biological processing steps until the mature miRNA, a 15–22 nt single-strand RNA, enters the RNA-protein complex known as the RNA-induced silencing complex (RISC), which contains an Argonaute (AGO) family protein that binds the single-stranded guide miRNA [3, 4]. When bound to target mRNA, the RISC complex mediates post-transcriptional silencing of mRNAs comprising sequences that are incompletely or fully complementary to the RISC-loaded miRNA [5]. Imperfect base pairing between miRNAs and mRNAs occurs frequently in mammalians, and enables an individual miRNA to simultaneously target the expression of a large cohort of mRNAs and thus to regulate a myriad of target proteins translated from such mRNAs.

miRNAs regulate a wide variety of biological processes, including tumourigenesis [6–9]. Over the last 10 years, it has been increasingly described that miRNAs are differentially expressed between normal and cancer cells, and that some miRNAs may act as tumour suppressors, while others as oncogenes, thus promoting tumour initiation and progression [10]. Altered miRNA expression can contribute, among others, to cellular de-differentiation, oncogenesis, metastasis, tumour invasion and angiogenesis [11].

The miR-143/miR-145 cluster is composed of two co-transcribed miRNAs, miR-143 and miR-145, which have distinct roles in cellular function [12, 13]. Both miR-143 and miR-145 are broadly described as downregulated in numerous solid tumours, including colon cancer [14]. The delivery of miR-143 and miR-145 in experimental cancer models appears to be beneficial as a potential cancer therapy [15, 16]. These miRNAs are involved in the regulation of several cellular processes including proliferation, migration and chemoresistance [17–19]. We have shown that miR-143 overexpression reduces colon tumour growth and proliferation *in vivo*, with increased apoptosis [20]. Similarly, miR-145 overexpression has been shown to regulate apoptosis [21] and to inhibit colon cancer cell proliferation [21, 22]. In colon cancer mouse xenografts, forced expression of miR-145 suppresses tumour growth [23] and angiogenesis [22]. In addition, combination of miR-143 and miR-145 overexpression inhibited colon cancer cell line proliferation and migration *in vitro*, decreasing *in vivo* tumour growth [24]. Importantly, we have previously shown that miR-143 acts as a sensitizer to 5-fluouracil [17], and either miR-143 or miR-145 induce tumour cell sensitization to cetuximab-mediated cellular cytotoxicity [25]. Together, these findings suggest that miR-143 and miR-145 play an important role in colon cancer onset and sensitization to anti-cancer therapy, which highlights their potential as a miRNA-based therapeutic approach.

In this study, comparative proteomic analysis of HCT116 colon cancer cells with stable miR-143 or miR-145 overexpression identified several differentially expressed proteins associated with the regulation of protein folding, cell death and response to oxidative stress. Particularly, expression of the antioxidant enzyme superoxide dismutase 1 (SOD1) was reduced in cells overexpressing miR-143 or miR-145. Further, miR-143 overexpression increased reactive oxygen species (ROS) generation, which was abrogated by reintroduction of SOD1. Our data also show that miR-143 and miR-145 reduced cell proliferation and migration and increased apoptosis in HCT116 cells treated with oxaliplatin. In miR-143 overexpressing cells this effect was triggered by ROS generation. Importantly, genetic and pharmacological approaches to inhibit oxidative stress rescued such effect. In recent years, the inhibition of ROS-scavenging mechanisms, with concomitant administration of pro-oxidizing agents, such as chemotherapy and/or radiotherapy, appears to be a potential therapeutic option to overcome resistance to cancer therapy [26]. Furthermore, blockage of antioxidant machinery has been shown to sensitize cancer cells to apoptotic cell death induced by further ROS production [27–29]. Therefore, increased oxidative stress generated by miR-143 might be an important mechanism to circumvent resistance of colon cancer cells to oxaliplatin.

## Materials and methods

### Cell culture

HCT116, HT29 and SW620 human colon carcinoma cell line was purchased from European Collection of Cell Cultures (ECACC) (Porton Down, Salisbury, UK) and cultured respectively in McCoy’s medium, Roswell Park Memorial Institute (RPMI) 1640 medium and Dulbecco’s modified Eagle’s medium (DMEM) supplemented with 10% foetal bovine serum (FBS) and 1% antibiotic/antimycotic solution. We generated miR-143 and miR-145 stably overexpressing HCT116 cells as previously described [25]. Briefly, HCT116 cells were transduced with retroviral particles carrying MSCV-Neo constructs overexpressing miR-143, miR-145 or Empty vector, followed by selection with 1 mg/ml neomycin. Cell lines were maintained at 37°C in a humidified atmosphere of 5% CO_2_. Cells were seeded at a density of 1.5 x 10^6^ cells/dish in 100 mm dishes for total proteomic analysis; 150 000 cells/well in 6-well plates for total protein and RNA extraction; 5 000 cells/well in 96-well plates for MTS, caspase-3/7 activity and ROS assays; 5 × 10^4^ cells/well in 12-well plates over 18 mm glass coverslips for nuclear morphology assay; 3 × 10^6^ cells/dish in 35 mm^3^ dishes for wound healing assay; and 25 000 cells/well in 24-well plates for Nexin assay.

### Cell culture and protein extraction for two-dimensional electrophoresis

HCT116 cells overexpressing miR-143 and miR-145, and Empty control cells, were seeded, allowed to grow for 72 h and processed for total protein extraction. Briefly, cells were washed with phosphate saline buffer (PBS), collected and lysed using ice-cold lysis buffer (50 mM Tris-HCl, pH 8, 150 mM NaCl, 5 mM EDTA) supplemented with 0.1% dithiothreitol (DTT), 1% Nonidet P-40, 1x Halt Protease and Phosphatase Inhibitor Cocktail (Thermo Fisher Scientific) for 30 min. Then, the lysates were centrifuged at 12,000 g for 10 min, at 4°C. Total proteins in the supernatant were recovered and stored at -80°C. Protein concentrations were determined using the Bio-Rad protein assay kit according to the manufacturer's instructions.

### Two-dimensional electrophoresis

For isoelectric focusing (IEF), 100 μL of the total crude extract was treated with 2-D Clean-Up Kit (GE Healthcare) for removal of IEF interfering molecules (e.g. lipids and salts), according to manufacturer’s instructions. Precipitated proteins were resuspended in 150 μL of rehydration buffer (2% (v/v) CHAPS (GE Healthcare), 7 M urea (GE Healthcare), 2 M thiourea (GE Healthcare), 1x protease inhibitors (cOmplete ULTRA Tablets, Mini, EDTA-free, EASYpack Protease Inhibitor Cocktail, Roche), 1x phosphatase inhibitors (PhosSTOP, Roche), 1 mM PMSF (GE Healthcare), 0.5% (w/v) DTT (GE Healthcare), 0.5% (v/v) Destreak rehydration solution (GE Healthcare), 0.5% (v/v) IPG electrolytes 3–10 NL (GE Healthcare), traces of bromophenol blue (GE Healthcare)), and incubated at room temperature for 2 h. After protein quantification using the Pierce 660 nm protein assay reagent (Thermo Scientific), samples with 200 μg of protein in a total volume of 125 μL of rehydration buffer were used to actively rehydrate Immobiline Drystrip (IPG strips, GE Healthcare) with 7 cm and 3–10 non-linear pH in an Ettan IPGphor instrument (GE Healthcare), followed by the IEF using a maximum voltage of 5000 V. The IPG strips were incubated for 15 min in equilibration buffer 1 (50 mM Tris-HCl pH 8.8, 2% (w/v) SDS, 6 M urea, 30% (v/v) glycerol, 1% (w/v) DTT and traces of bromophenol blue), followed by another incubation for 15 min in equilibration buffer 2 (50 mM Tris-HCl pH 8.8, 2% (w/v) SDS, 6 M urea, 30% (v/v) glycerol, 2.5% (w/v) iodoacetamide and traces of bromophenol blue), and placed on top of a 10% SDS-polyacrylamide gel for second dimension. After SDS-PAGE electrophoresis, gels were incubated in PhastGel Blue R-350 (GE Healthcare) according to manufacturer’s instructions. Gel images were acquired in an Image Scanner II from GE Healthcare. Gels were performed in duplicate for each condition.

### Analysis of protein expression levels

The analysis of gel images was performed using Melanie 7.0 software (Geneva Bioinformatics, Geneva, Switzerland). Protein spot detection was first performed automatically using the software, followed by manual confirmation. For protein expression analysis, we considered the volume of each individual spot normalized against the total volume of spots of the respective gel. The average of normalized volume of the matched protein spot in duplicated gels of the same condition was then used to calculate the fold variation between different conditions. The protein expression was altered if the fold between two conditions was lower than 0.7 (lower protein expression) or higher than 2 (increased protein expression).

### Protein spot identification

For identification of proteins of interest, reference maps published elsewhere were used [30, 31]. Additionally, to confirm the correct translations from the reference map and to identify other proteins of interest, protein spots were manually excised from the gel and identified in the UniMS–Mass Spectrometry Unit, ITQB/IBET (Oeiras, Portugal) using Peptide Mass fingerprint.

The groups of altered proteins between conditions were analysed in STRING (Search tool for the retrieval of interacting genes/proteins) database to infer possible protein-protein interactions, sub-cellular locations, and major altered pathways.

### Cell transfection and treatment

For SOD1, GRP94 and ANXA2 expression protein analysis, at the time of platting, cells were transfected with 40 nM of specific miR-143 or miR-145 precursors (premiR-143, AM17100; premiR-145, AM11480) alone or in combination, or with a pre-miR negative control (premiR-C, AM17110), using Lipofectamine 3000 (Thermo Fisher Scientific), according to the manufacturer’s protocol, and collected 72 h later. pCI-neo expression vector (pCI-neo) and p-CI-neo expression vector containing human wild-type SOD1 (pCI-neo-SOD1) were produced at Don Cleveland's lab (University of California, San Diego, USA), and kindly provided by Dora Brites (iMed.ULisboa, Lisbon, Portugal) [32]. At the time of platting, cells were transfected with the indicated plasmids using Lipofectamine 3000. Forty-eight hours after, total ROS measurement was performed and, in parallel, cells were collected to evaluate SOD1 expression levels. Oxaliplatin (kindly provided by Hospital Santa Maria, Lisbon, Portugal) and N-acetyl-L-cysteine (NAC; Sigma-Aldrich) stock solutions were prepared in PBS and stored at -20°C. Before treatments, cells were plated and allowed to adhere for 24 h. Cells were treated with 10 μM oxaliplatin or treated with oxaliplatin and exposed to 1mM NAC during 16 h for the evaluation of caspase-3/7 activity, during 48 h for apoptosis cell death assay, and during 24 h for total ROS measurement.

### Quantitative RT-PCR

Total RNA was extracted from cells with TRIzol reagent (Life Technologies), according to the manufacturer’s instructions. The expression of miR-143 and miR-145 was analysed by quantitative reverse transcription PCR (qRT-PCR) by TaqMan MicroRNA Reverse Transcription Kit and TaqMan MicroRNA assays for hsa-miR-143, hsa-miR-145, and human RNU6B for normalization to endogenous control. The relative amount of miR-143 or miR-145 was determined by the threshold cycle (2^−ΔΔCT^) method, where ΔΔCT = (CT_miRNA_−CT_RNU6B_) sample − (CT_miRNA_−CT_RNU6B_) calibrator. Real-time RT-PCR was performed in an Applied Biosystems 7300 System (Life Technologies), as previously described [17].

### Immunoblot analysis

Total protein extraction was performed as described previously [25]. Steady-state levels of ANXA2, GRP94 and SOD1 proteins were determined by immunoblot analysis. Briefly, 50 μg of total protein extracts were separated on 10 or 12% SDS-polyacrylamide electrophoresis gels and transferred onto nitrocellulose membranes. Membranes were blocked with 5% milk and incubated overnight with primary mouse antibodies reactive to GRP94 (#MABT829; Merck Millipore) and ANXA2 (#610068; BD Bioscience) or primary rabbit antibody reactive to SOD1 (#sc-11407; Santa Cruz Biotechnology). Membranes were then incubated with horseradish peroxidase-conjugated anti-mouse or anti-rabbit immunoglobulin secondary antibodies (1:5000 dilution; Bio-Rad). For protein detection, membranes were processed using Immobilon Western Chemiluminescent HRP Substrate (Merck Millipore) or Super Signal Femto Substrate (Pierce, Rockford, IL, USA) in a ChemiDoc MP System (Bio-Rad). β-actin (#A5541; Sigma-Aldrich) was used as loading control. The relative intensities of protein bands were quantified using the Image Lab densitometric analysis program (version 5.1; Bio-Rad).

### Cell proliferation

*In vitro* cell growth was assessed using the CellTiter 96 AQueous Non-Radioactive Cell Proliferation Assay (Promega, Madison, WI, USA), which is based on the bioreduction of the 3 (4, 5-dimethylthiazol-2-yl)-5-(3- carboxymethoxyphenyl)-2-(4-sulfophenyl)-2H-tetrazolium inner salt (MTS) to a water-soluble formazan product. Briefly, cell lines were seeded in 96-well plates and MTS metabolism was assayed at 4, 24, 48, 72 and 96 h after plating. For this purpose, 20 μL of MTS/PMS solution (19:1) was added to culture medium at the indicated time-points, and absorbance readings were measured at 490 nm, using a GloMax-Multi^+^ Detection System (Promega).

### Nuclear morphology evaluation

HCT116 cells stably expressing miR-143 or miR-145, and empty control cells, were seeded for 48 h. Then, attached cells were washed with PBS to remove detached cells, fixed with 4% paraformaldehyde in PBS for 20 min, washed with PBS, and stained with 5 μg/ml Hoechst 33258 (Sigma-Aldrich) in PBS for 15 min at room temperature, protected from light. Subsequently, coverslips were washed with PBS and mounted on glass slides with Fluoromount-G (Beckman Coulter, Inc., Brea, CA). Fluorescent nuclei were evaluated by fluorescence microscopy using a Zeiss Axio Scope.A1 fluorescence microscope (Carl Zeiss Microscopy GmbH), equipped with an AxioCam HRm (Carl Zeiss Microscopy GmbH).

### Wound healing assay

HCT116 cells with stable overexpression of miR-143 and miR-145 and empty control cells, were seeded and allowed to grow to confluence. Next, “wounds” were performed using a sterile 10-μL tip, followed by 2 washes with culture medium. Cell migration to the “wound area” was evaluated and images captured at 24, 48, and 72 h with a Zeiss Primo Vert microscope (Carl Zeiss Microscopy GmbH, Jena) connected to a Leica DFC 40 camera (Leica Microsystems AG, Heersbrugg).

### Caspase-3/7 activity assay

Caspase-3 and -7 activation status was measured using the Caspase-Glo 3/7 Assay (Promega). For this propose, 75 μL of Caspase-Glo 3/7 reagent was added to each well, plates were mixed by orbital shaking for 30 s, and incubated at room temperature for 30 min. The resulting luminescence was measured using the GloMax-Multi+ Detection System (Promega).

### Apoptosis evaluation

Apoptotic cell death was quantified using the Guava Nexin Reagent kit (Merck Millipore). Briefly, cell culture supernatant and adherent cells were collected, centrifuged and re-suspended in PBS containing 2% FBS for incubation with Guava Nexin Reagent. Next, 50 μl of cell suspension were mixed with 50 μl of Guava Nexin Reagent, incubated for 20 min at room temperature, protected from light, and assayed promptly, using a Guava easyCyte 5HT flow cytometer (Merck Millipore). Sample acquisition and analysis were performed using the InCyte software module (Merck Millipore).

### Total ROS measurement

For total ROS measurement, cells were incubated for 40 min at 37°C, 5% CO_2_, with 10 μM 2′,7′-dichlorodihydrofluorescein diacetate (H2DCFDA; Sigma-Aldrich Co.), a cell-permeant nonfluorescent molecule that is oxidized by ROS, resulting in the accumulation of a fluorescent compound (dichlorofluorescein). Cells were washed twice with PBS 1X and the emission of green fluorescence measured using the GloMax-Multi+ Detection System (Promega). Total protein concentration was used for normalization purposes.

### Statistical analysis

Data are expressed as mean ± SEM from at least three independent experiments. Statistical analysis was performed using GraphPad Prism version 6.00 software. One-way ANOVA test with Turkey’s post-test was used for selected comparisons when more than two groups were analysed and Student’s *t*-test when two groups were analysed. Values of *p* < 0.05 were considered significant. Principal Component Analysis was performed using XLStats 2014 software. A significant protein-protein interaction was considered when PPI enrichment *p*-value was *p* < 0.001 and False Discovery Rate of the group of proteins belonging to the same GO pathway was *q*-value < 0.001.

## Results

### Identification of differentially expressed proteins in miR-143 or miR-145 overexpressing cells

Protein extracts from HCT116 human colon cancer cells stably overexpressing miR-143 or miR-145, and from HCT116 Empty control cells (S1 Fig) were subjected to proteomic analysis to hint at molecular players involved in colon cancer control by miR-143 and miR-145. Proteomic analysis was based on two-dimensional electrophoresis (2-DE) focused on the most abundant proteins with isoelectric points ranging between 3 and 10. Protein spots were identified by Maldi-TOF Mass Spectrometry and comparison with a reference map of HCT116 cells proteome prepared under identical conditions [30, 31] (S2 Fig). One-way ANOVA test with Turkey’s post-test analysis for comparison between the 3 conditions (HCT116 Empty cells, HCT116 overexpressing miR-143 cells, and HCT116 overexpressing miR-145 cells) retrieved a *p*-value < 0.0001.

**Fig 1:**
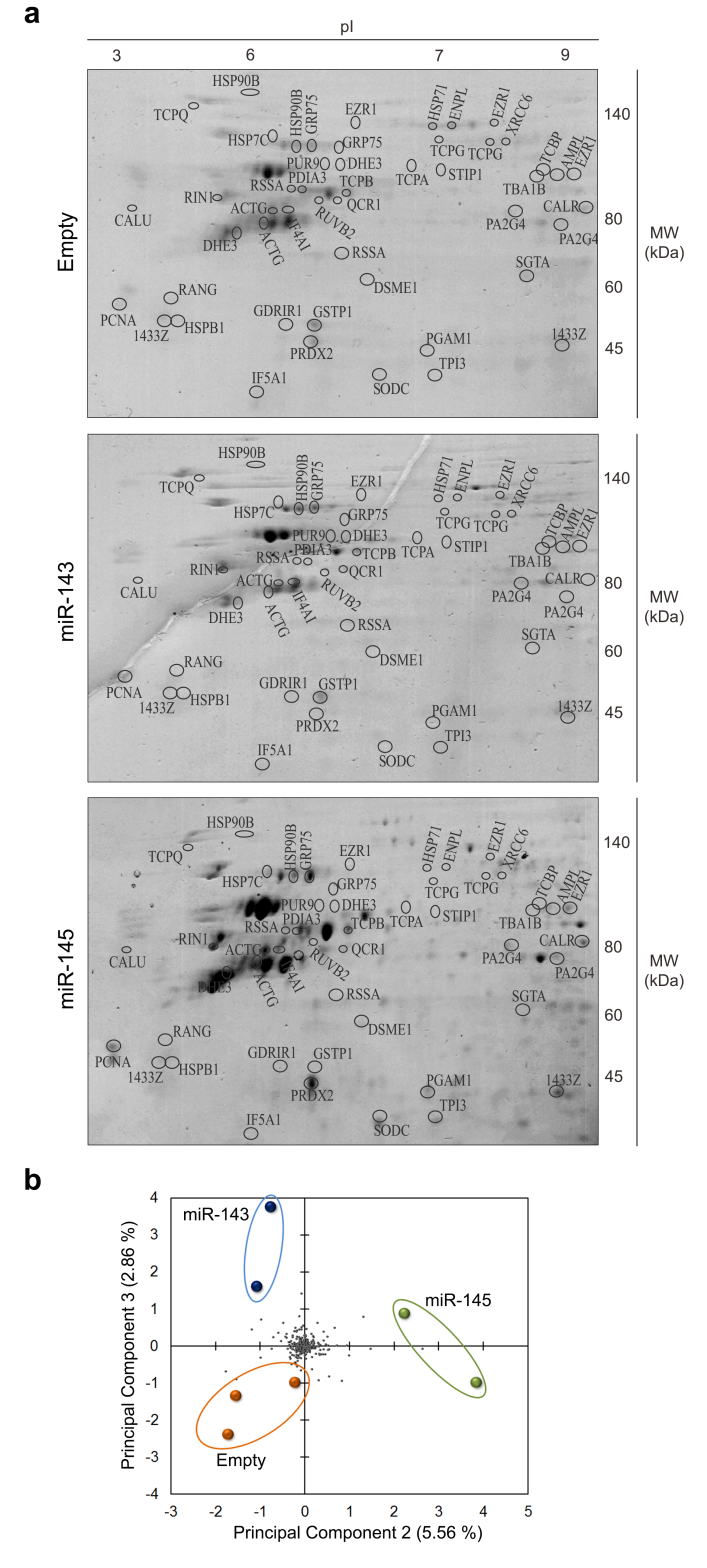
Two-dimensional gel electrophoresis analysis of HCT116 human colon cancer cells overexpressing miR-143, miR-145, or Empty vector. Proteins of HCT116 stably transduced cells were separated by 2-DE (IEF (pI 3–10 non-linear) + SDS-PAGE) and visualized by staining with Coomasie brilliant blue R-350. (**a**) Representative 2-DE gels. Identified proteins are listed in S1 and S2 Tables. (**b**) Principal Component Analysis (PCA). Coloured spots represent each gel prepared for the indicated condition. Grey spots represent proteins detected in the 2-DE gels.

Forced expression of miR-143 or miR-145 in HCT116 cells resulted in increased proteins in the 2-DE patterns. While 211 protein spots were identified in the control, 244 and 349 proteins were identified in HCT116 cells overexpressing miR-143 and miR-145, respectively (Fig 1A). The volume percentage of the proteins identified in each sample is described in S1 Table. Moreover, 50 protein spots were present only in the 2-DE protein patterns of cells overexpressing miR-143, and 142 spots were identified only in cells overexpressing miR-145.

To identify the differences between protein patterns across samples, the fold variation between HCT116 cells overexpressing miR-143, miR-145 or Empty vector control was calculated (S2 Table). A variation was considered substantial if the fold-change was < 0.7 (underexpressed) or > 2 (overexpressed) compared to Empty vector control cells, and a *p*-value < 0.05 (except for a few cases where a tendency of alteration in proteins belonging to the same biological process was observed) [30, 31]. A gross analysis of protein expression variation between samples showed that approximately 30% of proteins displayed altered abundance both in miR-143 and miR-145 overexpressing cells, from a total of 83 differentially expressed proteins in miR-143 overexpressing *versus* Empty cells and 112 in miR-145 overexpressing *versus* Empty cells. From the total altered proteins, showing a differential fold expression < 0.7 or > 2 between each test sample and the control, we identified 45 proteins in miR-143 overexpressing cells, and 48 proteins in miR-145 overexpressing cells (S2 Table). Finally, a principal component analysis (PCA) showed that each protein 2-DE pattern obtained for each condition has distinct variances, and revealed that the variation between protein patterns was more pronounced in cells overexpressing miR-145 (Fig 1B).

### Gene ontology and pathway enrichment analysis of altered proteins

Proteins identified that showed a differential expression between each sample and the control were subjected to analysis using STRING, functional protein network database version 10.0 (http://string-db.org, accessed in March, 2017). Protein-protein interaction network analysis revealed a significant interaction between 35 proteins in miR-143 overexpressing HCT116 cells (S3A Fig), and 40 proteins in miR-145 overexpressing HCT116 cells (S3B Fig and Table 1) (*p* < 0.001).

**Table 1:** GO biological processes altered in miR-143 or miR-145 overexpressing HCT116 human colon cancer cells compared to Empty control cells with False Discovery Rate < 0.05.

GO Pathway ID	Pathway description	Number of proteins	
		miR-143	miR-145
GO:0006457	Protein folding	10	11
GO:0051084	*De novo* posttranslational protein folding	5	7
GO:0000302	Response to reactive oxygen species	6	6
GO:0042981	Regulation of apoptotic process	11	13

Results from analysis on STRING, functional protein network database version 10.0 (http://string-db.org/, accessed in March 2017). Only pathways common to miR-143 or miR-145 overexpressing HCT116 cells are indicated.

**Fig 2:**
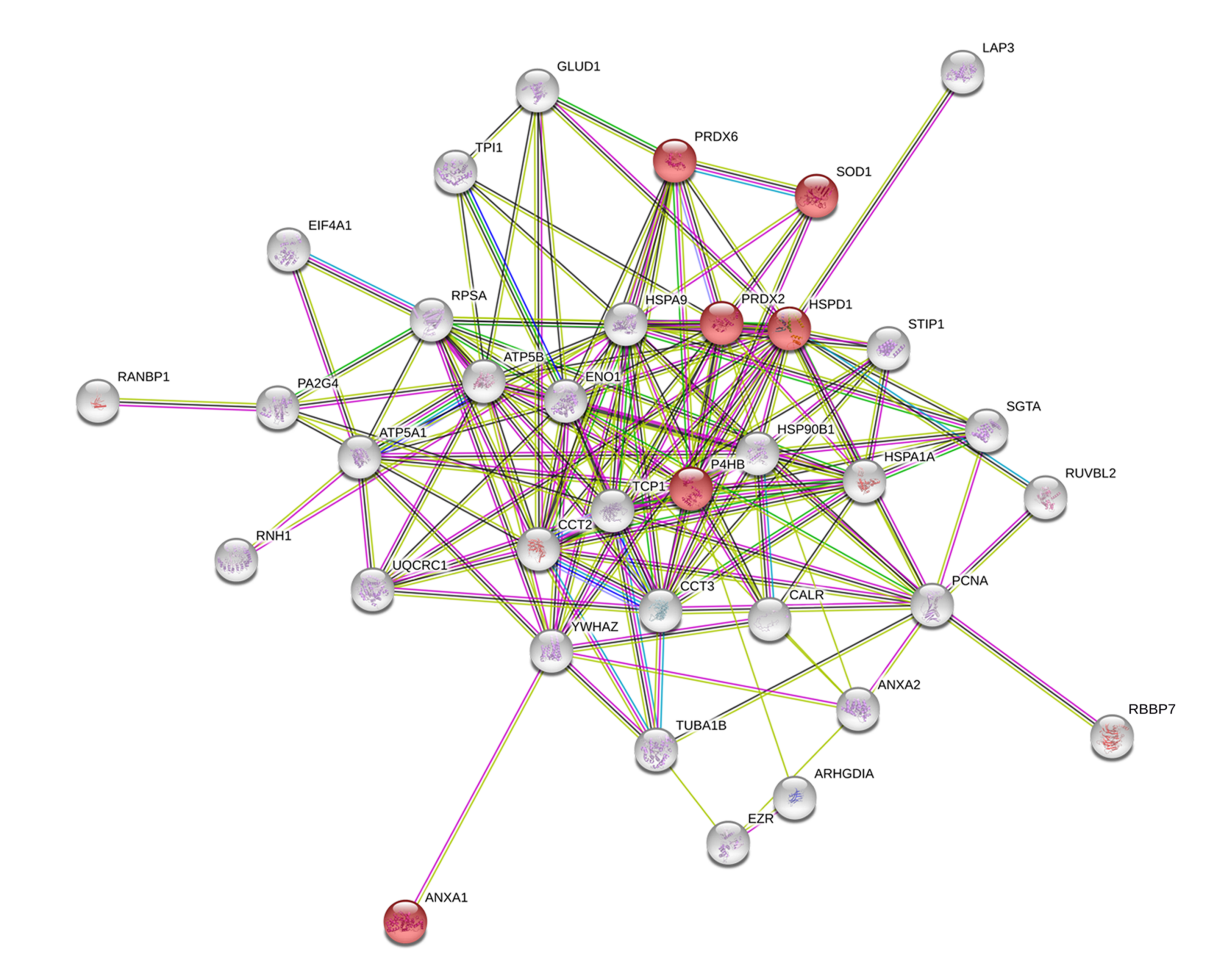
Protein-protein network with altered expression in HCT116 human colon cancer cells overexpressing miR-143 relative to Empty vector. Nodes represent proteins and lines connecting nodes indicate direct, or indirect interactions between proteins. Red nodes represent proteins involved in response to ROS (Biological Process GO pathway number 0000302).

Among the interacting proteins in miR-143 overexpressing cells, 31% of these proteins are involved in the regulation of apoptotic processes (*q*-value < 0.001) (Biological Process GO pathway number 0042981), including superoxide dismutase [Cu-Zn] (SODC/SOD1), peroxiredoxin-2 (PRDX2), peroxiredoxin-6 (PRDX6), heat shock protein 90 kDa beta member 1 (HSP90B1/ENPL/GRP94), T-complex protein 1 subunit alpha (TCP1), Rho GDP-dissociation inhibitor 1 (GDIR1/ARHGDIA) and calrecticulin (CALR), and annexin II (ANXA2). Moreover, within the protein-protein network obtained for miR-143 overexpressing cells, a group of 6 proteins was identified, and found to be involved in the response to ROS (*q*-value < 0.001) (Biological Process GO pathway number 0000302), including SOD1, PRDX2, PRDX6, ANXA1, underexpressed in miR-143 overexpressing cells relative to Empty cells, and P4HB and HSPD1, overexpressed in miR-143 overexpressing cells relative to Empty cells (Fig 2). Interestingly, SOD1, PRDX2 and PRDX6 belong to the cellular antioxidant machinery (molecular function GO: pathway number 0016209).

In miR-145 overexpressing HCT116 cells, 35% of the identified interacting proteins are involved in the regulation of cell death (*q*-value < 0.001) (GO Pathway number 0010941), including proliferation-associated protein 2G4 (PA2G4), pyrroline-5-carboxylate reductase 1, mitochondrial (P5CR1), T-complex protein 1 subunit alpha (TCPA), heat shock proteins HSPD1, HSP90B1, HSP90AB1, HSPA9 and HSPB1, protein disulphide isomerase (PDIA1), Rho GDP-dissociation inhibitor 1 (GDIR1), glutathione S-transferase (GSTP1), peroxideroxin-2 (PRDX2), calreticulin (CALR), and the proteasome activator complex subunit 1 (PSME1) (S3B Fig).

The KEGG pathway enrichment analysis revealed considerable enrichment of proteins involved in processing in the endoplasmic reticulum (*q*-value < 0.001), pathways in cancer (*q*-value < 0.001), and miRNAs in cancer (*q*-value < 0.001) (restricted to miR-143 overexpression).

### Validation of differentially expressed proteins in miR-143 or miR-145 overexpressing cells

Our results showed that proteins found to be significantly modulated in miR-143 or miR-145 overexpressing cells are involved in cell death pathways, response to oxidative stress and protein processing in endoplasmic reticulum. We selected SOD1, GRP94 and ANXA2 to validate differential protein expression and evaluated steady state levels by immunoblotting in cells with stable or transient miR-143 or miR-145 overexpression (S1 Fig).

**Fig 3:**
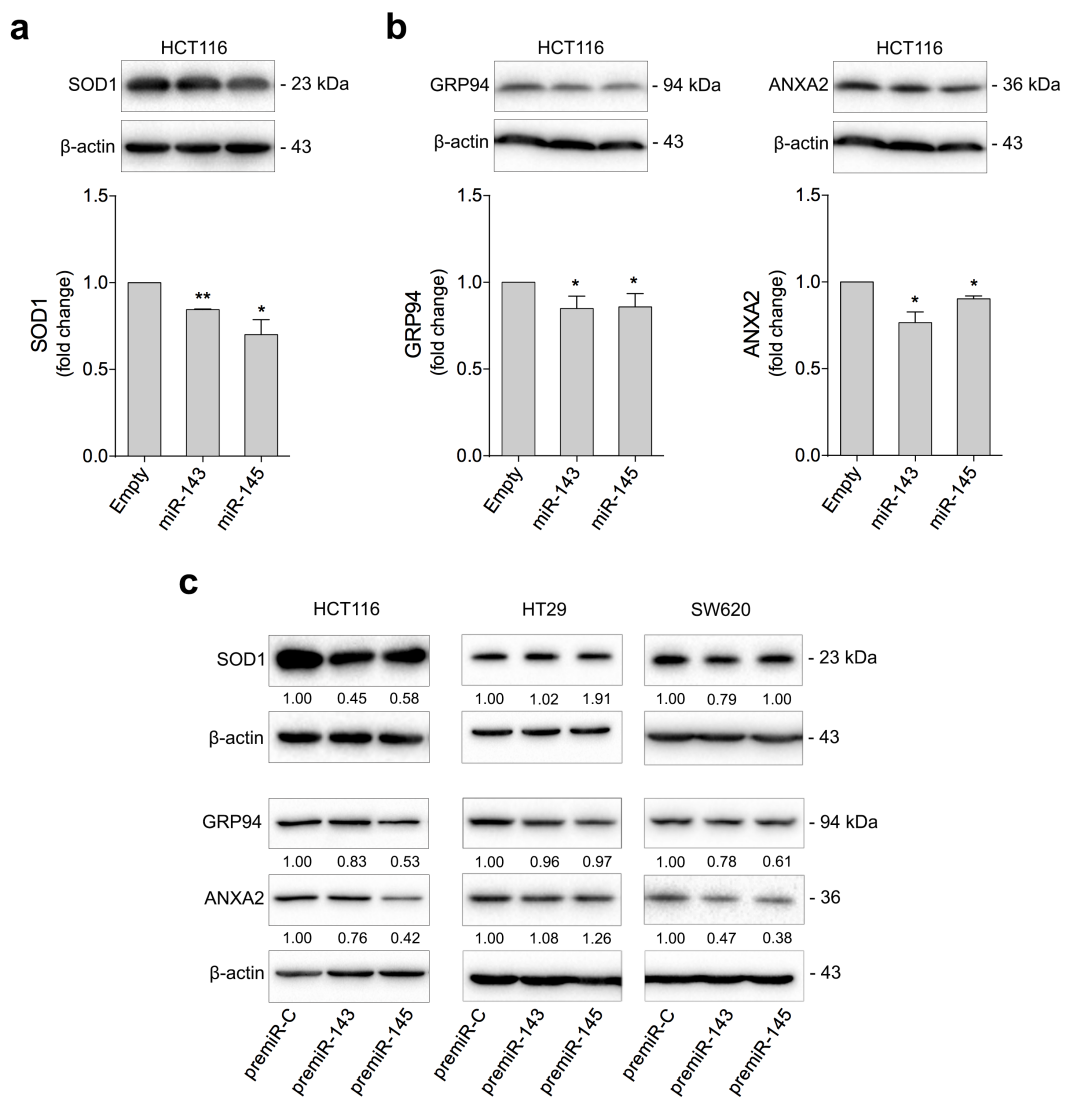
miR-143 and miR-145 overexpression reduce SOD1, GRP94 and ANXA2 protein expression in human colon cancer cells. HCT116 cells were stably transduced with miR-143, miR-145, or Empty vector. HCT116, HT29 and SW620 cells were transiently transfected with miR-143 (premiR-143), miR-145 (premiR-145), or control (premiR-C) precursors. Cells were plated for protein expression analysis. (**a**) SOD1, (**b**) GRP94 and ANXA2 protein expression levels were analysed by immunoblotting (top) in HCT116 stably transduced cells. (**c**) SOD1, GRP94 and ANXA2 protein expression levels were also analysed in HCT116, HT29 and SW620 transiently transfected cells. Representative blots are shown. Results are expressed as mean ± SEM fold change to control cells, from at least three independent experiments. ***p < 0.001, **p < 0.01, *p < 0.05 from control cells.

SOD1 is a cytosolic superoxide dismutase that converts superoxide to hydrogen peroxide (H_2_O_2_), maintaining low levels of superoxide in the cytosol, thus protecting cells from oxidative stress and cell death. Furthermore, SOD1 has been identified as a novel cancer drug target, suggesting that SOD1 inhibition impairs cancer [33–35]. Consistent with the proteomic data, SOD1 was downregulated in HCT116 cells with stable miR-143 or miR-145 overexpression compared to control cells, as confirmed by Western blotting (*p* < 0.05) (Fig 3A, top).

In turn, GRP94 and ANXA2 steady-state levels were reduced in HCT116 cells with stable miR-143 or miR-145 overexpression (*p* < 0.05) (Fig 3B).

Protein expression was also evaluated in HCT116, HT29 and SW620 cells with transient miR-143 or miR-145 overexpression (Fig 3C). HCT116 and SW620 cells followed the same trend with reduced levels of the relevant proteins. HT29 cells, with different genetic background, showed relatively unchanged expression levels of the same proteins.

### miR-143-induced oxidative stress is abrogated by SOD1 overexpression

Oxidative stress is described as an imbalance between the production of ROS and their elimination by the cellular antioxidant machinery. Indeed, cancer cells overproduce ROS as by-products of their increased metabolism to drive and sustain growth. As high levels of ROS can induce cell death [36], cancer cells display increased levels of intracellular antioxidant proteins to overcome ROS stress and allow ROS stimulation of pro-tumourigenic signalling, without causing cell death [37]. Therefore, inhibition of antioxidant proteins may render cancer cells more susceptible to cell death induction.

**Fig 4:**
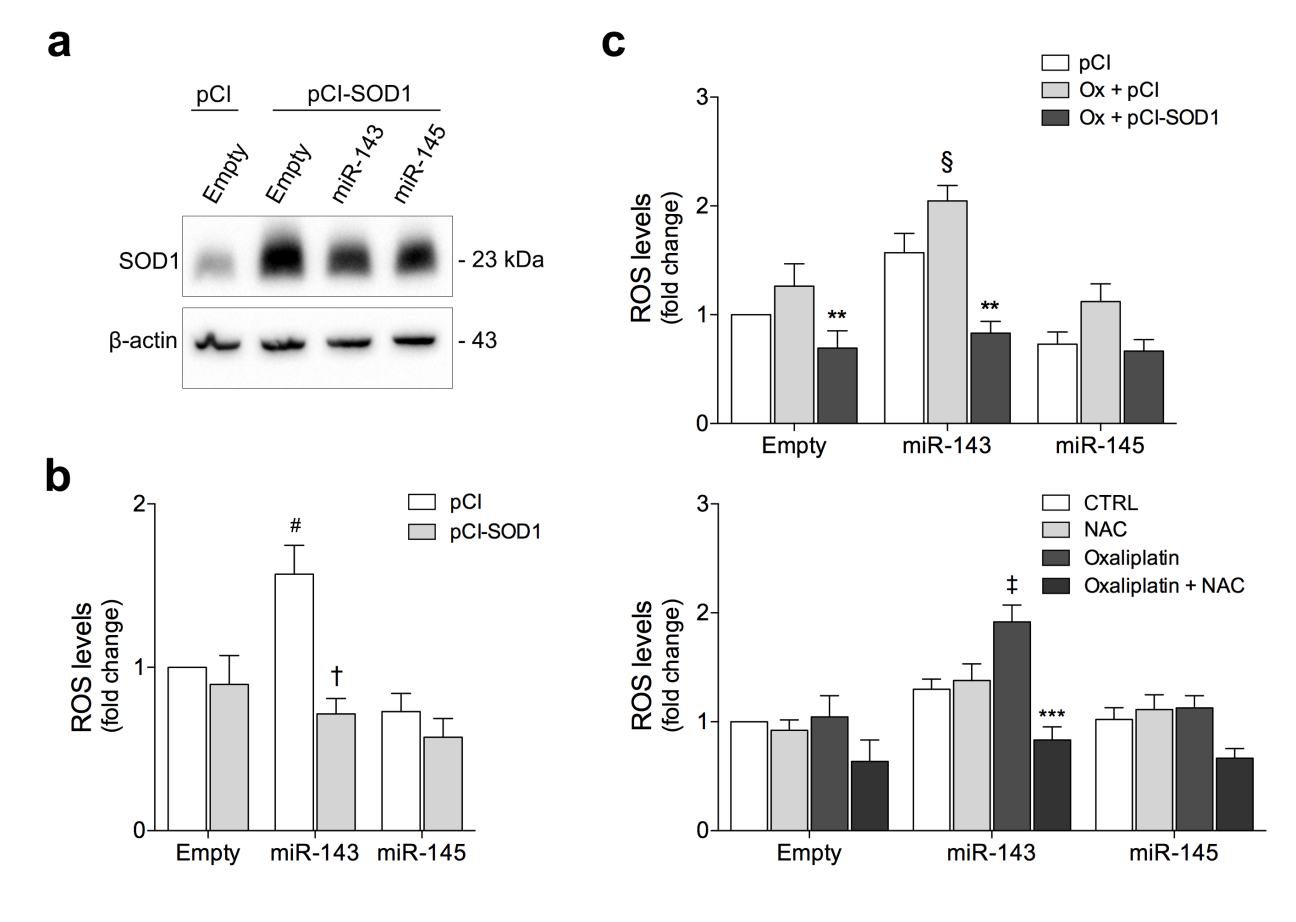
SOD1 gain-of-function and NAC exposure counteract miR-143-induced oxidative stress in HCT116 human colon cancer cells. HCT116 cells stably transduced with miR-143, miR-145, or Empty vector were transfected with pCI-neo (pCI) or pCI-neo-SOD1 (pCI-SOD1) plasmids, or exposed to NAC and treated with oxaliplatin (Ox). (**a**) SOD1 steady-state levels were analysed by immunoblotting. (**b**) ROS levels were determined by DCFH staining in SOD1 overexpressing cells. (**c**) ROS levels were determined by DCFH staining in cells treated with oxaliplatin (Ox) overexpressing SOD1 (top) or exposed to NAC (bottom). Representative blots are shown. Results are expressed as mean ± SEM fold change to Empty control cells, from at least three independent experiments. ^#^*p* < 0.05 from Empty cells transfected with pCI-neo construct; ^†^*p* < 0.01 from respective pCI-neo transfected cells; ^§^*p* < 0.05 and ^‡^*p* < 0.01 from Empty cells treated with oxaliplatin; **p < 0.01 and ***p < 0.001 from respective oxaliplatin treated cells.

Our functional proteomic data suggested that increased levels of miR-143 regulate response to oxidative stress, consistent with reduced levels of the antioxidant enzymes SOD1, PRDX2 and PRDX6 in these cells (Fig 2 and S2 Table). To further investigate the role of SOD1 in miR-143 or miR-145-induced oxidative stress, we transfected cells with either a vector overexpressing human wild-type SOD1 or the respective control vector (Fig 4A). Next, we evaluated the endogenous production of ROS in cells overexpressing miR-143 or miR-145 (Fig 4B). Cells with miR-143 overexpression display increased ROS production compared to Empty cells (*p* < 0.05). Interestingly, re-introduction of SOD1 completely abrogated miR-143-induced ROS production (*p* < 0.01). No significant effect was observed in miR-145 overexpressing cells.

In agreement with these results, treatment with the platinum-based agent oxaliplatin, known to generate high levels of ROS, promote DNA damage and induce apoptosis [38–40] resulted in a 2-fold increase in intracellular ROS levels in miR-143 overexpressing cells, compared to Empty-treated cells (*p* < 0.05) (Fig 4C, top). Importantly, forced SOD1 expression decreased oxaliplatin-induced oxidative stress (*p* < 0.01) (Fig 4C, top). In line with these observations, pharmacological inhibition of ROS with antioxidant NAC abrogated ROS production in miR-143 overexpressing cells treated with oxaliplatin (*p* < 0.001) (Fig 4C, bottom).

### miR-143 and miR-145 impact on cell proliferation and morphology and sensitize to oxaliplatin-induced apoptosis

**Fig 5:**
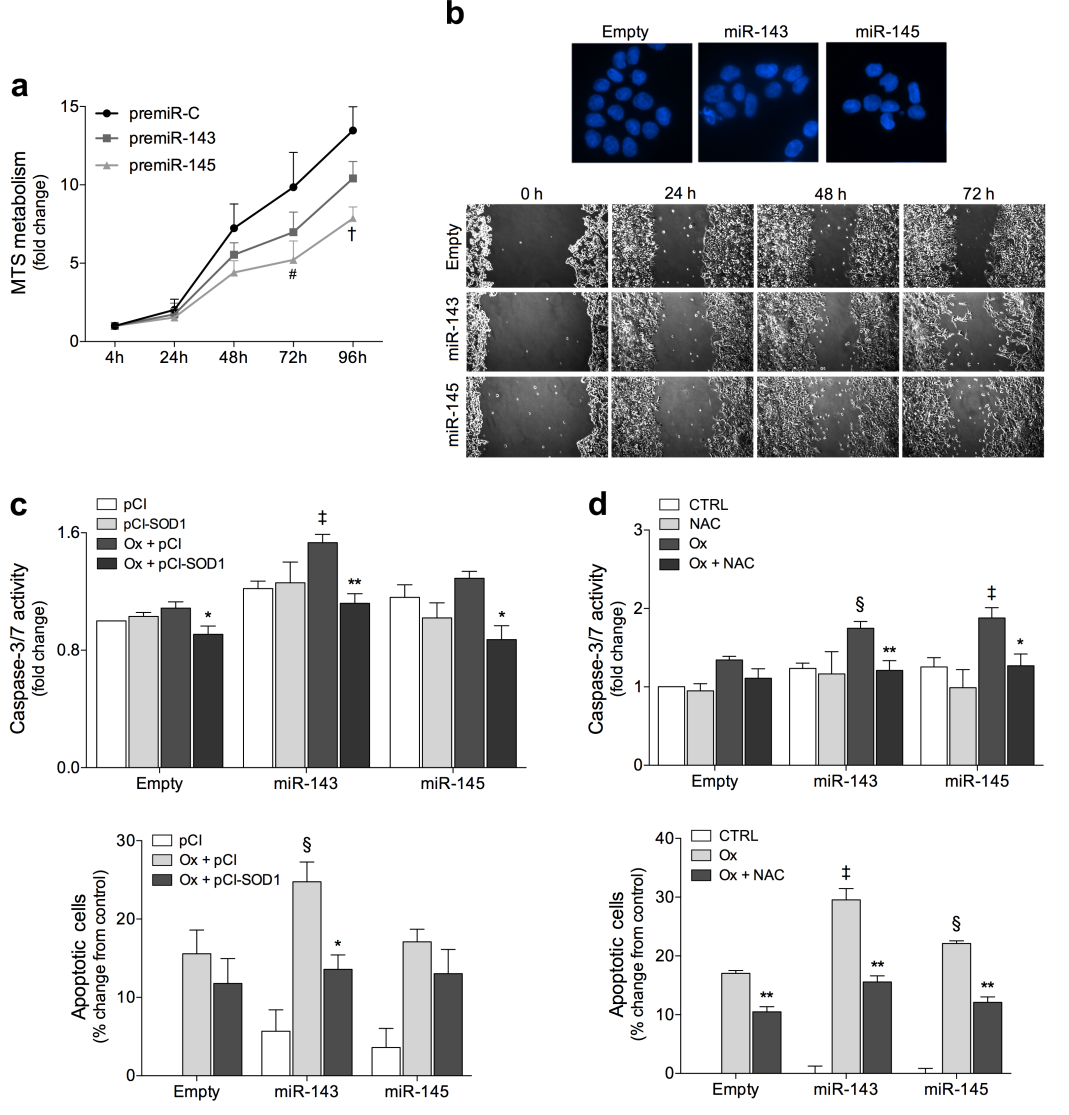
miR-143 and miR-145 impact on cell proliferation and morphology and miR-143-induced oxidative stress contributes to oxaliplatin-mediated apoptosis in HCT116 human colon cancer cells. (**a**) The growth profiles of HCT116 cells transiently expressing miR-143 (premiR-143), miR-145 (premiR-145), or control (premiR-C) precursors were monitored by MTS metabolism assay at 4, 24, 48, 72 and 96 h after plating. (**b**) Nuclear morphology was evaluated by fluorescence microscopy after Hoechst staining. Representative images of Hoechst staining at 400x magnification are presented (top). Cell migration was assessed by wound healing assay at 24, 48 and 72 h after wound formation. Representative images are presented (bottom). HCT116 cells stably transduced with miR-143, miR-145, and Empty vector were treated with oxaliplatin (Ox) and transfected with pCI-neo (pCI) or pCI-neo-SOD1 (pCI-SOD1) plasmids (**c**), or exposed to NAC (**d**). Caspase-3/7 activity was determined by Caspase-Glo 3/7 assay. Apoptosis was quantified by flow cytometry using Guava Nexin assay. Results are expressed as mean ± SEM fold change and percentage change of apoptotic cells ± SEM, from at least three independent experiments. ^#^*p* < 0.05 and ^†^*p* < 0.01 from control precursor (premiR-C) transfected cells; ^‡^*p* < 0.01; ^§^*p* < 0.05 from Empty cells treated with oxaliplatin; ***p < 0.001, **p < 0.01, *p < 0.05 from respective oxaliplatin treated cells.

Functional proteomic analysis also indicated that regulation of apoptosis might be a biological process regulated by both miR-143 and miR-145. Subsequently, we investigated the effects of miR-143 and miR-145 overexpression in colon cancer cell proliferation. Cell growth profiles showed that miR-143 and miR-145 overexpression reduced HCT116 cell proliferation by up to 20 and 40% (*p* < 0.01) at 96 h, respectively, compared to empty vector control cells (Fig 5A). In addition, overexpression of miR-143 or miR-145 enhanced nuclear fragmentation (Fig 5B, top), and decreased cell migration (Fig 5B, bottom), as compared to empty control cells.

Next, to investigate whether the overexpression of miR-143 and miR-145 would contribute to oxaliplatin-induced apoptosis, we performed caspase-3/7 activity assays and flow cytometry analysis of annexin V and 7-AAD staining. After oxaliplatin treatment, miR-143 and miR-145 overexpression increased caspase-3/7 activity as well as apoptotic cells (Fig 5C and 5D). In addition, SOD1 overexpression partially blocked cell death induced by oxaliplatin in miR-143 overexpressing cells, as shown by caspase activity (*p* < 0.01) (Fig 5C, top) and annexin V/7-AAD (*p* < 0.05) (Fig 5C, bottom) assays. NAC exposure, in turn, decreased apoptosis induced by miR-143 or miR-145 overexpression and oxaliplatin treatment, as shown by a significant reduction in caspase activity (*p* < 0.05) (Fig 5D, top) and in apoptotic cells (*p* < 0.01) (Fig 5D, bottom). Caspase-3/7 activity and annexin V/7-AAD assays also showed that combined overexpression of miR-143 and miR-145 did not induce further changes after oxaliplatin treatment as compared with overexpression of individual miRNAs (S4 Fig).

## Discussion

Aberrant miRNA expression is associated with deregulation of signalling pathways, which in turn cause abnormal cell growth and de-differentiation, leading and contributing to cancer. Our understanding of the molecular mechanisms underlying the intricate role of miRNAs in cellular signalling remains incomplete. Proteome-based approaches can assess protein expression patterns and its regulation by miRNAs. Using bioinformatics tools, it is also possible to explore the pathways that are modulated by specific miRNAs.

In this study, using two-dimension gel electrophoresis and mass spectrometry we analysed protein expression patterns of HCT116 colon cancer cells overexpressing either miR-143 or miR-145. Proteomic analysis revealed a total of 83 differentially expressed proteins in miR-143 overexpressing cells and 112 in miR-145 overexpressing cells. Protein-protein interaction network analysis identified main pathways regulated by miRNAs, including cell death, response to oxidative stress and protein folding pathways. SOD1, GRP94 and ANXA2 proteins are among those whose steady state levels were downregulated by miR-143 or miR-145 overexpression. Similar results were observed in SW620 cells. In HT29 cells, the effects were less pronounced, which could be explained by the KRAS status. Both HCT116 and SW620 cells but not HT29 cells display a mutation in the *KRAS* oncogene. Since KRAS acts early in transducing signals from EGFR, mutations in this gene can recapitulate the regulatory instructions transmitted by activated EGFR. Also, the EGFR signalling pathway suppresses miR-143/145 in colonic cells, while overexpression of these miRNAs suppresses EGFR-induced colon cancer cell growth [41]. In prostate cancer, oncogenic KRAS signalling led to suppression of miR-143/145 cluster by activating Ras-responsive element-binding protein 1 (REBB1). In turn, KRAS and RREB1 are miR-143 and miR-145 direct targets, respectively, potentiating KRAS-mediated tumourigenesis [42]. In colon cancer cell lines, overexpression of these miRNAs down-regulates KRAS, RREB1 and other genes in the MAPK cascade, which abrogate signalling through MAPK, PI3K and JNK pathways [43]. HCT116 and SW620 cells may thus be more susceptible to miR-143 and miR-145 effects. More studies are needed to further evaluate these effects.

SOD1 belongs to the family of copper/zinc superoxide dismutase enzymes, and converts the superoxide into less reactive H_2_O_2_, thereby protecting the cell from oxidative stress-induced injury and consequent cell death. Increased generation of ROS and cellular oxidant stress has been reported as strongly associated with malignant transformation, metastasis and resistance to anticancer therapy. As a consequence of genetic, metabolic and microenvironment-associated changes, tumour cells exhibit persistently high levels of ROS. To face oxidative stress conditions, these cells have developed a proficient mechanism of ROS detoxification, which sustains survival under pro-oxidizing conditions. Therefore, cancer cells are highly dependent on their antioxidant machinery to maintain the redox balance, and very vulnerable to agents that increase oxidative stress above the toxicity threshold. Inhibition of antioxidant systems effectively kills cancer cells, sparing normal cells, due to their lower intracellular ROS levels [26, 37, 44]. Thereby, specific inhibitors of the antioxidant enzymes, such as SOD1, have been identified as potential therapeutic approaches and are currently in phase II clinical studies. In fact, SOD1 inhibition using a highly specific copper-binding compound induced antiangiogenic effects, proliferation inhibition and tumour apoptotic cell death [27, 45, 46]. SOD1, although not predicted as a direct target of miR-143 or miR-145 in bioinformatics analysis (miRanda MiRBase, Target Scan and MiRDB), was reduced after miR-143 and miR-145 overexpression in human colon cancer cells, thus suggesting indirect targeting. Importantly, our results showed that ROS levels in miR-143 overexpressing cells, but not in miR-145 overexpressing cells, were greater than in control cells, and re-introducing SOD1 abrogated miR-143-induced ROS production. Although miR-143 and miR-145 have targets in common, they play distinct roles in cellular function. This is demonstrated in cardiomyocytes, in which miR-143 has been reported to be increased under oxidative stress conditions, with miR-143 inhibition rescuing cells from oxidative stress-induced apoptosis [47]. Forced expression of miR-145 resulted in inhibition of ROS production and oxidative stress-induced apoptosis [48]. Therefore, SOD1 reduction in miR-145 overexpressing cells may not be enough to increase oxidative stress, as other important targets regulating ROS production in these cells may also play a role.

Mechanisms of miR-143 and miR-145 ROS-mediated cell death in cancer are not fully explored. Therefore, to better understand the involvement of these miRNAs in oxidative stress-induced cell death in colon cancer cells, we evaluated the levels of ROS in miR-143 and miR-145 overexpressing cells treated with the chemotherapeutic drug oxaliplatin. Oxaliplatin is a third-generation platinum-based agent used as first line treatment for patients with colon cancer [49]. Platinum-based agents induce intra- and interstrand DNA cross-links and platinum-DNA adducts [50], and generate high levels of ROS and toxic oxygen metabolites that induce DNA damage and apoptosis [38–40]. Our data demonstrate that miR-143 overexpression induces high ROS generation in cells treated with oxaliplatin, which translates into a functional increase in apoptosis. Others have also reported that miR-143 overexpression sensitizes colon cancer cells to oxaliplatin through classical caspase-3-dependent apoptosis [19]. In this regard, our results strongly suggest that enhanced oxidative stress generated by miR-143 might be an important mechanism to sensitize colon cancer cells to the cytotoxic effects of oxaliplatin. Further reinforcing this, inhibition of oxidative stress by genetic (SOD1 overexpression) or pharmacological (NAC) approaches prevented oxaliplatin-induced apoptosis.

Accumulating evidence shows that miR-143 reduces anti-apoptotic protein Bcl-2 [17], and sensitizes cells to Fas-induced apoptosis [51]. Likewise, miR-145 has been shown to induce caspase-dependent apoptosis in colon cancer by targeting DNA fragmentation factor (DFF45) [21], and to sensitize cells to tumour necrosis factor-related apoptosis-inducing ligand (TRAIL)-induced apoptosis [52, 53]. In addition, both miR-143 and miR-145 reduce cell proliferation and migration, and enhanced apoptosis during cetuximab-dependent cellular cytotoxicity by increasing caspase-3/7 activity and PARP cleavage, and by reducing Bcl-2 protein levels [25]. In this regard, we show here that miR-145 overexpression also induces apoptosis in HCT116 cells treated with oxaliplatin, suggesting that miR-145 enhances chemosensitivity of cancer cells to oxaliplatin treatment. We found that miR-145 overexpression in HCT116 cells is not associated with an increase in ROS generation. However, oxidative stress inhibition in miR-145 overexpressing cells following oxaliplatin treatment significantly reduced caspase-3/7 activity, while NAC exposure partially blocked apoptosis, suggesting that oxidative stress-induced apoptosis may be induced by oxaliplatin treatment, but may not be the major cell death mechanism at play in miR-145 overexpressing cells. Further studies are required to investigate the role of miR-145 in chemosensitization of HCT116 colon cancer cells to oxaliplatin induced cell death.

## Conclusion

This study identified differentially expressed proteins in miR-143 and miR-145 overexpressing cells using proteomic analysis, further validated for three candidate proteins related to carcinogenesis. SOD1, in turn, was inversely correlated with miR-143-induced oxidative stress. Moreover, miR-143 and miR-145 sensitized colon cancer cells to oxaliplatin treatment by increasing apoptosis. In miR-143 overexpressing cells, ROS generation and the resulting oxidative stress mediate oxaliplatin-induced apoptosis. This highlights the chemosensitizing effects of miR-143 in colon cancer cells, reinforcing its potential to circumvent resistance to anticancer therapy. A miRNA mimetic of miR-34 has become the first miRNA to enter clinical trial in oncology [54] further suggesting that restoring tumour-suppressive miRNAs might be beneficial as an anti-cancer therapeutic approach.

## Supporting information

S1 Fig **miR-143 and miR-145 expression in HCT116 (a, b), HT29 (c), and SW620 (b) human colon cancer cells.** (**a**) Cells were stably transduced with MSCV-Neo construct expressing either miR-143, miR-145, or Empty vector. (**b, c** and **d**) Cells were transiently transfected with specific precursors of miR-143 (premiR-143), miR-145 (premiR-145), alone or together, or miRNA negative control (premiR-C). miRNA expression was assayed by Taqman real-time PCR. Results are expressed as mean ± SEM fold change to control cells. ***p < 0.001, **p < 0.01 from control cells.
(PDF)

S2 Fig 2-DE proteome map of HCT116 human colon cancer cells. Proteins were separated by IEF (pI 3–10 non-linear) in the first dimension and SDS-PAGE in the second dimension, and visualized by staining with Coomasie brilliant blue R-350.
(PDF)

S3 Fig Protein-protein network in HCT116 human colon cancer cells overexpressing miR-143 or miR-145, relative to Empty vector. Nodes represent proteins and lines connecting nodes indicate direct or indirect interactions between proteins. (**a**) Protein-protein network altered in HCT116 cells overexpressing miR-143. Red nodes represent proteins involved in the regulation of apoptotic processes (Biological Process GO: 0042981). (**b**) Protein-protein network altered in HCT116 cells overexpressing miR-145. Red nodes represent proteins involved in the regulation of cell death (Biological Process GO:0010941). Red arrows represent proteins that were down-regulated in miR-143 or miR-145 2-DE patterns, while green arrows represent proteins that were up-regulated in miR-143 or miR-145 2-DE patterns.
(PDF)

S4 Fig miR-143 and miR-145 overexpression increases sensitivity to oxaliplatin-mediated apoptosis in human colon cancer cells. HCT116 cells transiently transfected with miR-143 (premiR-143), miR-145 (premiR-145), or control (premiR-C) precursors were treated with oxaliplatin (Ox). Caspase 3/7 activity was determined using Caspase-Glo 3/7 assay (left). Apoptosis was quantified by flow cytometry using Guava Nexin assay (right). Results are expressed as mean caspase activity ± SEM fold change and percentage change of apoptotic cells ± SEM, from at least three independent experiments. ***p < 0.001, **p < 0.01, *p < 0.05 from Empty cells treated with oxaliplatin.
(PDF)

S1 Table Average and standard deviation of the % volume of proteins in at least three independent 2-DE maps of HCT116 human colon cancer cells stably overexpressing miR-143, miR-145 or Empty vector. (PDF)

S2 Table Fold variance of common proteins between HCT116 human colon cancer cells overexpressing miR-143, miR-145 or Empty vector. (PDF)
