# Supplementary material for: Convergence of miR-143 overexpression, oxidative stress and cell death in HCT116 human colon cancer cells
Source: PLoS One. 2018 Jan 23;13(1):e0191607. doi: 10.1371/journal.pone.0191607 (PMC5779689; doi:10.1371/journal.pone.0191607)
Supplement: S1 Fig — miR-143 and miR-145 expression in HCT116 (a, b), HT29 (c), and SW620 (b) human colon cancer cells. (a) Cells were stably transduced with MSCV-Neo construct expressing either miR-143, miR-145, or Empty vector. (b, c and d) Cells were transiently transfected with specific precursors of miR-143 (premiR-143), miR-145 (premiR-145), alone or together, or miRNA negative control (premiR-C). miRNA expression was assayed by Taqman real-time PCR. Results are expressed as mean ± SEM fold change to control cells. ***p < 0.001, **p < 0.01 from control cells. (PDF) [file pone.0191607.s001.pdf]

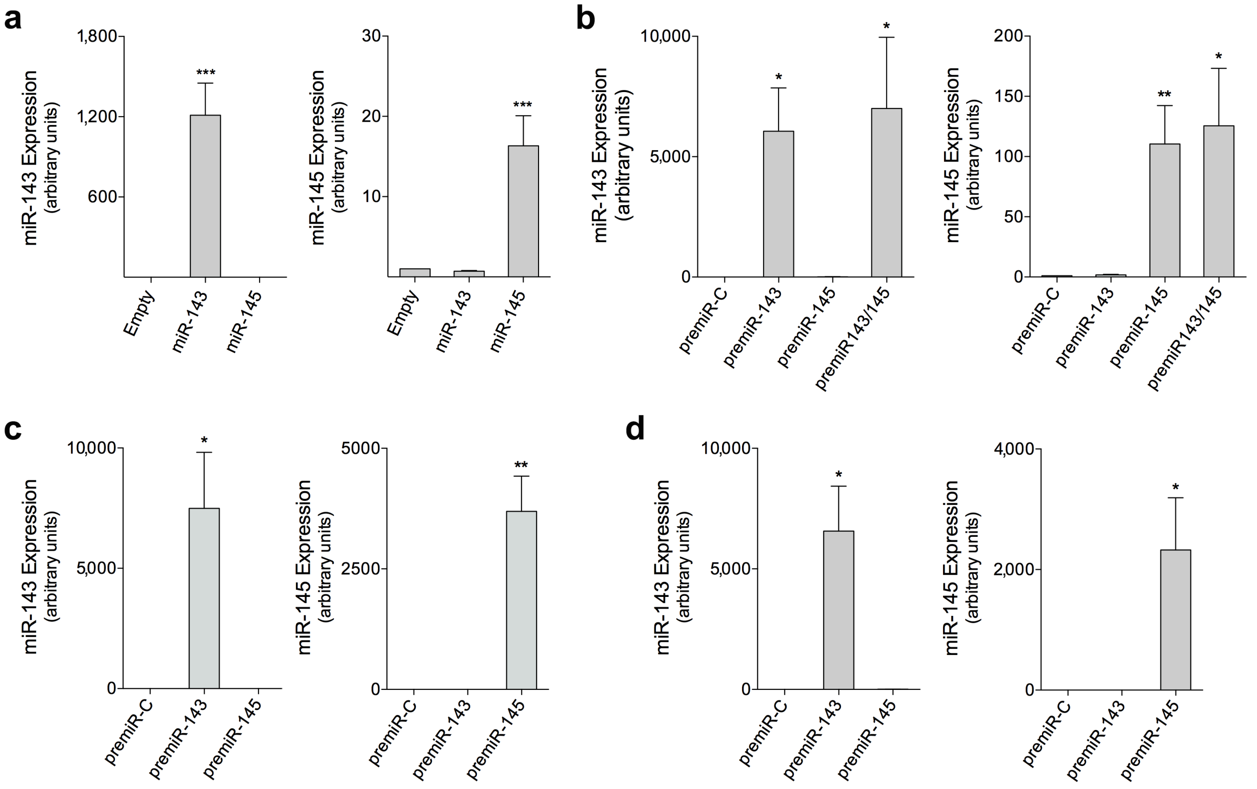


**S1 Fig. miR-143 and miR-145 expression in HCT116 (a, b), HT29 (c), and SW620 (b) human colon cancer cells.** (**a**) Cells were stably transduced with MSCV-Neo construct expressing either miR-143, miR-145, or Empty vector. (**b, c** and **d**) Cells were transiently transfected with specific precursors of miR-143 (premiR-143), miR-145 (premiR-145), alone or together, or miRNA negative control (premiR-C). miRNA expression was assayed by Taqman real-time PCR. Results are expressed as mean ± SEM fold change to control cells. ****p* < 0.001, ***p* < 0.01 from control cells.
