# Supplementary material for: Convergence of miR-143 overexpression, oxidative stress and cell death in HCT116 human colon cancer cells
Source: PLoS One. 2018 Jan 23;13(1):e0191607. doi: 10.1371/journal.pone.0191607 (PMC5779689; doi:10.1371/journal.pone.0191607)
Supplement: S2 Fig — Proteins were separated by IEF (pI 3–10 non-linear) in the first dimension and SDS-PAGE in the second dimension, and visualized by staining with Coomasie brilliant blue R-350. (PDF) [file pone.0191607.s002.pdf]

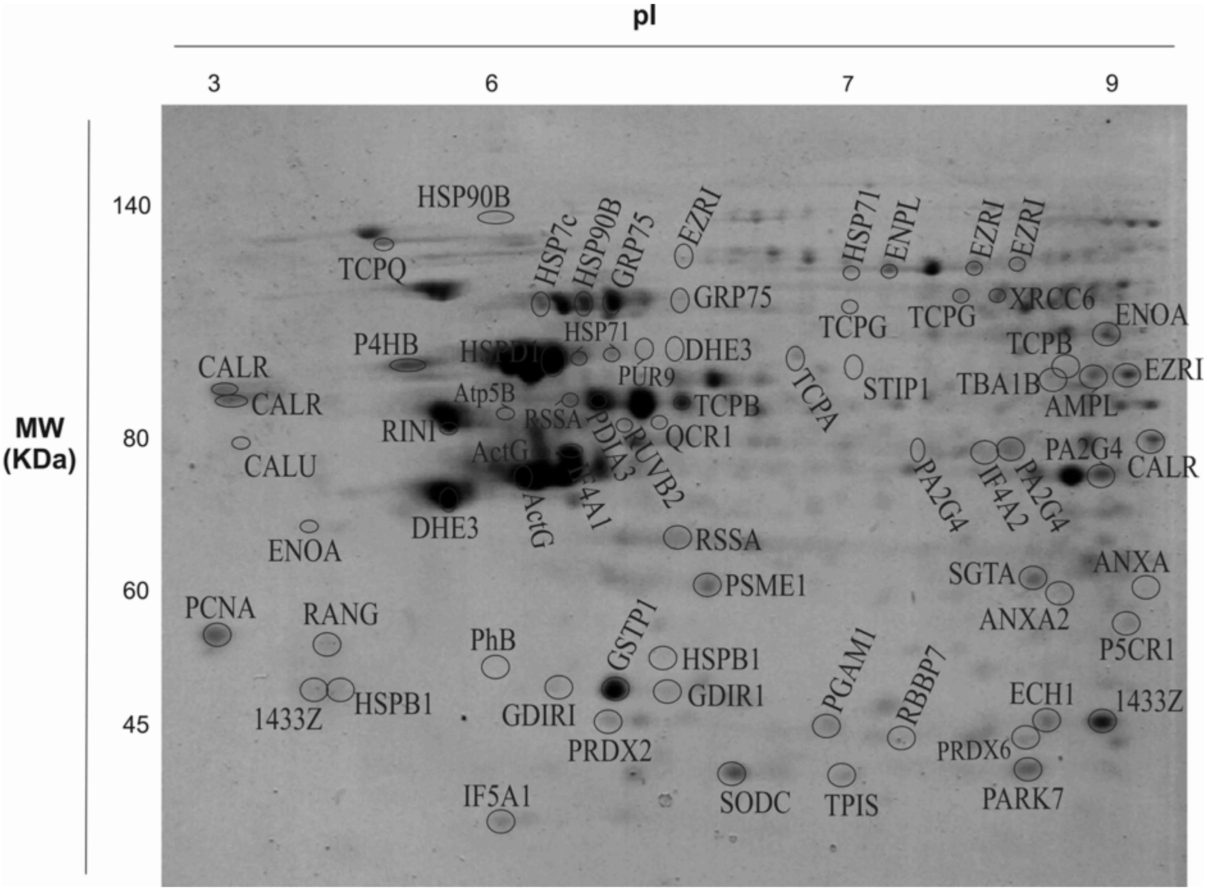

S2 Fig. 2-DE proteome map of HCT116 human colon cancer cells. Proteins were separated by IEF (pI 3-10 non-linear) in the first dimension and SDS-PAGE in the second dimension, and visualized by staining with Coomasie brilliant blue R-350.
