# Supplementary material for: Convergence of miR-143 overexpression, oxidative stress and cell death in HCT116 human colon cancer cells
Source: PLoS One. 2018 Jan 23;13(1):e0191607. doi: 10.1371/journal.pone.0191607 (PMC5779689; doi:10.1371/journal.pone.0191607)
Supplement: S3 Fig — Nodes represent proteins and lines connecting nodes indicate direct or indirect interactions between proteins. (a) Protein-protein network altered in HCT116 cells overexpressing miR-143. Red nodes represent proteins involved in the regulation of apoptotic processes (Biological Process GO: 0042981). (b) Protein-protein network altered in HCT116 cells overexpressing miR-145. Red nodes represent proteins involved in the regulation of cell death (Biological Process GO:0010941). Red arrows represent proteins that were down-regulated in miR-143 or miR-145 2-DE patterns, while green arrows represent proteins that were up-regulated in miR-143 or miR-145 2-DE patterns. (PDF) [file pone.0191607.s003.pdf]

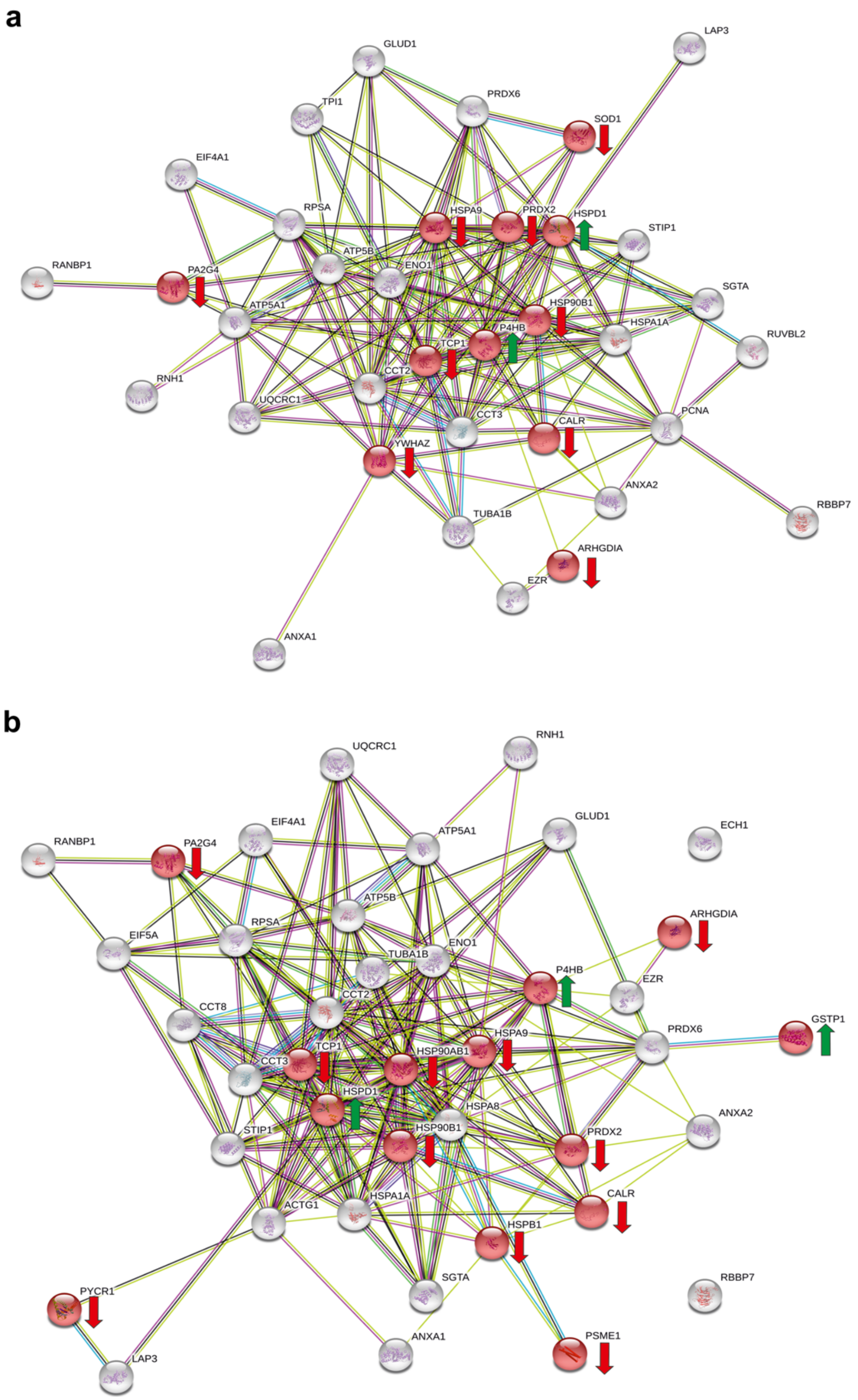

S3 Fig. Protein-protein network in HCT116 human colon cancer cells overexpressing miR-143 or miR-145, relative to Empty vector. Nodes represent

proteins and lines connecting nodes indicate direct or indirect interactions between proteins. (**a**) Protein-protein network altered in HCT116 cells overexpressing miR-143. Red nodes represent proteins involved in the regulation of apoptotic processes (Biological Process GO: 0042981). (**b**) Protein-protein network altered in HCT116 cells overexpressing miR-145. Red nodes represent proteins involved in the regulation of cell death (Biological Process GO:0010941). Red arrows represent proteins that were down-regulated in miR-143 or miR-145 2-DE patterns, while green arrows represent proteins that were up-regulated in miR-143 or miR-145 2-DE patterns.
