# Supplementary material for: Convergence of miR-143 overexpression, oxidative stress and cell death in HCT116 human colon cancer cells
Source: PLoS One. 2018 Jan 23;13(1):e0191607. doi: 10.1371/journal.pone.0191607 (PMC5779689; doi:10.1371/journal.pone.0191607)
Supplement: S4 Fig — HCT116 cells transiently transfected with miR-143 (premiR-143), miR-145 (premiR-145), or control (premiR-C) precursors were treated with oxaliplatin (Ox). Caspase 3/7 activity was determined using Caspase-Glo 3/7 assay (left). Apoptosis was quantified by flow cytometry using Guava Nexin assay (right). Results are expressed as mean caspase activity ± SEM fold change and percentage change of apoptotic cells ± SEM, from at least three independent experiments. ***p < 0.001, **p < 0.01, *p < 0.05 from Empty cells treated with oxaliplatin. (PDF) [file pone.0191607.s004.pdf]

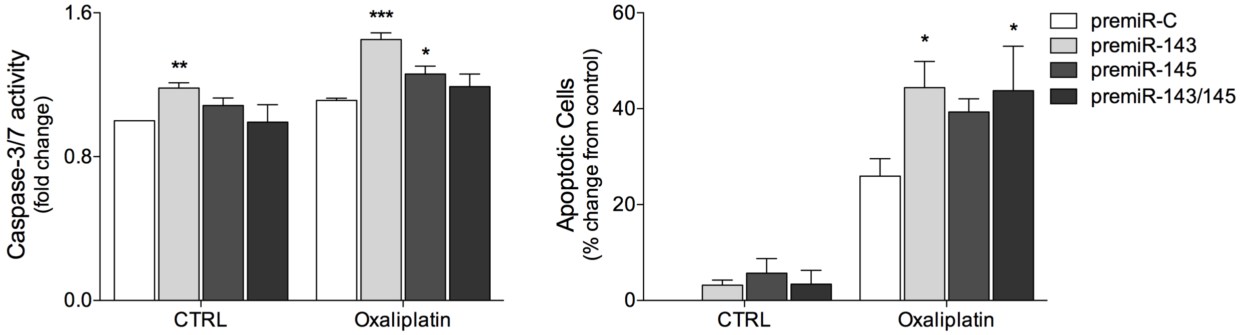

S4 Fig. miR-143 and miR-145 overexpression increases sensitivity to oxaliplatin-mediated apoptosis in human colon cancer cells. HCT116 cells transiently transfected with miR-143 (premiR-143), miR-145 (premiR-145), or control (premiR-C) precursors were treated with oxaliplatin (Ox). Caspase 3/7 activity was determined using Caspase-Glo 3/7 assay (left). Apoptosis was quantified by flow cytometry using Guava Nexin assay (right). Results are expressed as mean caspase activity ± SEM fold change and percentage change of apoptotic cells ± SEM, from at least three independent experiments. $^{***}p < 0.001$, $^{**}p < 0.01$, $^{*}p < 0.05$ from Empty cells treated with oxaliplatin.
