# Supplementary material for: Convergence of miR-143 overexpression, oxidative stress and cell death in HCT116 human colon cancer cells
Source: PLoS One. 2018 Jan 23;13(1):e0191607. doi: 10.1371/journal.pone.0191607 (PMC5779689; doi:10.1371/journal.pone.0191607)
Supplement: S1 Table — (PDF) [file pone.0191607.s005.pdf]

S1 Table. Average and standard deviation of the % volume of proteins in at least three independent 2-DE maps of HCT116 human colon cancer cells stably overexpressing miR-143, miR-145 or Empty vector.

Acession name_HUMAN	Empty		miR-143		miR-145	
	Mean	SD	Mean	SD	Mean	SD
TCPQ_HUMAN	1.19	0.64	1.11	0.33	0.33	0.03
HSP90B_HUMAN	0.32	0.08	0.41	0.10	0.24	0.01
HSP90B_HUMAN	1.02	0.16	1.19	0.04	0.71	0.04
ENPL_HUMAN	0.58	0.11	0.27	0.12	0.20	0.04
SGTA_HUMAN	0.41	0.05	0.25	0.11	0.07	0.02
1433Z_HUMAN	0.93	0.29	1.09	0.24	0.68	0.14
GSTP1_HUMAN	0.76	0.21	0.71	0.15	1.21	0.32
PSME1_HUMAN	0.31	0.30	0.37	0.12	0.19	0.02
IF5A1_HUMAN	0.39	0.10	0.28	0.08	0.70	0.10
PCNA_HUMAN	0.49	0.08	0.82	0.79	0.46	0.16
DHE3_HUMAN	0.49	0.47	1.00	0.19	2.01	0.80
ActG_HUMAN	0.49	0.53	0.47	0.28	0.95	0.32
ActG_HUMAN	0.49	0.31	0.47	0.11	0.41	0.26
IF4A1_HUMAN	0.49	0.07	0.23	0.10	0.34	0.15
RINI_HUMAN	0.49	0.06	0.89	0.25	0.17	0.01
Atp5B_HUMAN	0.49	0.22	0.23	0.02	0.17	0.10
CH60_HUMAN	0.49	0.55	2.71	0.05	1.49	0.17
HS71A_HUMAN	0.49	0.09	1.00	0.05	0.67	0.59
CALR_HUMAN	0.49	0.17	0.31	0.11	0.43	0.03
CALR_HUMAN	0.49	0.19	0.38	0.20	0.47	0.24
Atp5B_HUMAN	0.49	0.09	0.32	0.18	0.43	0.11
PDIA1_HUMAN	0.49	0.24	1.15	0.17	1.52	0.37
hsp7c_HUMAN	0.49	0.09	0.51	0.11	0.29	0.16
HS71A_HUMAN	0.49	0.12	0.95	0.28	0.23	0.20
GRP75_HUMAN	0.49	0.18	1.08	0.19	1.56	0.59
GRP75_HUMAN	0.49	0.14	0.71	0.36	0.43	0.04
PDIA3_HUMAN	0.49	0.09	0.51	0.04	0.63	0.40
RSSA_HUMAN	0.49	0.14	1.49	1.00	0.57	0.13
RUVB2_HUMAN	0.49	0.08	0.29	0.14	0.49	0.12
TCPB_HUMAN	0.49	0.20	0.55	0.04	0.46	0.00
GRP75_HUMAN	0.49	0.16	0.33	0.22	0.21	0.00
TCPA_HUMAN	0.49	0.10	0.14	0.08	0.10	0.02
HS71A_HUMAN	0.49	0.04	0.10	0.08	0.33	0.26
EZRI_HUMAN	0.49	0.02	0.19	0.09	0.03	0.01
EZRI_HUMAN	0.49	0.28	0.80	0.10	0.24	0.15

TCPG_HUMAN	0.49	0.15	0.16	0.10	0.04	0.01
STIP1_HUMAN	0.49	0.13	0.09	0.01	0.08	0.01
TCPG_HUMAN	0.49	0.23	0.16	0.05	0.22	0.06
XRCC6_HUMAN	0.49	0.04	0.38	0.01	0.37	0.07
EZRI_HUMAN	0.49	0.01	0.11	0.03	0.13	0.05
ENOA_HUMAN	0.49	0.11	0.47	0.18	0.11	0.03
EZRI_HUMAN	0.49	0.04	0.46	0.20	0.29	0.03
CALR_HUMAN	0.49	0.22	0.34	0.21	0.32	0.11
PA2G4_HUMAN	0.49	0.07	0.31	0.18	0.32	0.10
AMPL_HUMAN	0.49	0.23	0.32	0.16	0.25	0.08
TCBP_HUMAN	0.49	0.03	0.08	0.01	0.07	0.05
TBA1B_HUMAN	0.49	0.04	0.14	0.12	0.03	0.02
ATPA_HUMAN	0.49	0.05	0.09	0.01	0.14	0.01
QCR1_HUMAN	0.49	0.19	0.33	0.03	0.29	0.09
PA2G4_HUMAN	0.49	0.19	0.76	0.03	0.25	0.04
ENOA_HUMAN	0.49	0.18	0.54	0.34	0.55	0.15
ANXA_HUMAN	0.49	0.13	0.21	0.11	0.22	0.04
ENOA_HUMAN	0.49	0.34	0.36	0.01	0.45	0.03
ANXA2_HUMAN	0.49	0.28	0.15	0.10	0.15	0.04
P5CR1_HUMAN	0.49	0.18			0.30	0.09
ECH1_HUMAN	0.49	0.01	0.41	0.19	0.22	0.04
PRDX6_HUMAN	0.49	0.04	0.12	0.06	0.28	0.03
RBBP7_HUMAN	0.49	0.03	0.27	0.17	0.12	0.03
SODC_HUMAN	0.5	0.0	0.3	0.1	0.4	0.0
PRDX2_HUMAN	0.5	0.3	0.3	0.2	0.3	0.1
TPIS_HUMAN	0.5	0.1	0.3	0.1	0.5	0.1
GDIR1_HUMAN	0.49	0.05	0.21	0.06	0.19	0.12
GDIR1_HUMAN	0.5	0.0	0.1	0.0	0.4	0.2
RSSA_HUMAN	0.5	0.1	0.4	0.1	0.3	0.0
ENOA_HUMAN	0.49	0.09	0.24	0.03	0.20	0.01
1433Z_HUMAN	0.5	0.2	0.3	0.1	0.4	0.1
RANG_HUMAN	0.5	0.1	0.7	0.6	0.3	0.2
HSPB1_HUMAN	0.5	0.1	0.4	0.1	0.1	0.1
PGAM1_HUMAN			0.4	0.1	0.5	0.1
PhB_HUMAN			0.2	0.1	0.1	0.1
CALU_HUMAN					0.5	0.2
HSPB1_HUMAN					0.2	0.0
Park7_HUMAN					0.2	0.1
IF4A2_HUMAN					0.08	0.04
PA2G4_HUMAN					0.07	0.03
PUR9_HUMAN					0.1	0.0
DHE3_HUMAN					0.1	0.0

Results are shown as mean ± standard deviation (SD).
