# Supplementary material for: Convergence of miR-143 overexpression, oxidative stress and cell death in HCT116 human colon cancer cells
Source: PLoS One. 2018 Jan 23;13(1):e0191607. doi: 10.1371/journal.pone.0191607 (PMC5779689; doi:10.1371/journal.pone.0191607)
Supplement: S2 Table — (PDF) [file pone.0191607.s006.pdf]

S2 Table. Fold variance of common proteins between HCT116 human colon cancer cells overexpressing miR-143, miR-145 or Empty vector.

Uniprot Kb	Acession name_HUMAN	Gene	Name	miR-143		miR-145	
				miR-143 *vs* Empty	*p* value	miR-145 *vs* Empty	*p* value
P50990	TCPQ_HUMAN	CCT8	T-complex protein 1 subunit theta	0.94	0.89	0.27	0.17
P08238	HSP90B_HUMAN	HSP90AB1	Heat shock protein HSP 90 - beta	1.28	0.33	0.76	0.29
P08238	HSP90B_HUMAN	HSP90AB1	Heat shock protein HSP 90 - beta	1.18	0.24	0.70	0.09
P14625	ENPL_HUMAN	HSP90B1	Endoplasmin	0.46	0.05	0.34	0.02
O43765	SGTA_HUMAN	SGTA	Small glutamin-rich tetratricopeptide repeat-containing protein alpha	0.60	0.09	0.16	0.00
P63104	1433Z_HUMAN	YWHAZ	14-3-3 protein zeta/delta	1.17	0.58	0.73	0.36
P09211	GSTP1_HUMAN	GSTP1	Glutathione S-transferase P	0.94	0.80	1.58	0.15
Q06323	PSME1_HUMAN	PSME1	Proteasome activator complex subunit 1	1.19	0.81	0.60	0.61
P63241	IF5A1_HUMAN	EIF5A	Eukaryotic translation initiation factor 5A-1	0.72	0.28	1.78	0.05
P12004	PCNA_HUMAN	PCNA	Proliferating cell nuclear antigen	1.67	0.49	0.95	0.82
P00367	DHE3_HUMAN	GLUD1	Glutamate dehydrogenase 1, mitochondrial	2.05	0.11	4.10	0.77
P63261	ActG_HUMAN	ACTG1	Actin, cytoplasmic 2	0.96	0.34	1.94	1.00
P63261	ActG_HUMAN	ACTG1	Actin, cytoplasmic 2	0.96	0.32	0.84	0.30
P60842	IF4A1_HUMAN	EIF4A1	Eukaryotic initiation factor 4A-1	0.48	0.05	0.70	0.26
Q13671	RINI_HUMAN	RNH1	Ras and Rab interactor 1	1.81	0.18	0.34	0.00
P06576	Atp5B_HUMAN	ATP5B	ATP synthase subunit beta, mitochondrial	0.47	0.72	0.35	0.52
P10809	CH60_HUMAN	HSPD1	60 kDa heat shock protein, mitochondrial	5.54	0.99	3.03	0.06
P0DMV8	HS71A_HUMAN	HSPA1A	Heat shock 70 kDa protein 1A	2.05	0.79	1.36	0.40
P27797	CALR_HUMAN	CALR	Calreticulin	0.63	0.91	0.87	0.37
P27797	CALR_HUMAN	CALR	Calreticulin	0.77	0.68	0.96	0.42
P06576	Atp5B_HUMAN	ATP5B	ATP synthase subunit beta, mitochondrial	0.65	0.37	0.89	0.07
P07237	PDIA1_HUMAN	P4HB	Protein disulfide-isomerase	2.35	0.14	3.11	0.06

P11142	hsp7c_HUMAN	HSPA8	Heat shock cognate 71 kDa protein	1.03	0.24	0.59	0.48
P0DMV8	HS71A_HUMAN	HSPA1A	Heat shock 70 kDa protein 1A	1.95	0.07	0.48	0.20
P38646	GRP75_HUMAN	HSPA9	Stress-70 protein, mitochondrial	2.20	0.61	3.19	0.34
P38646	GRP75_HUMAN	HSPA9	Stress-70 protein, mitochondrial	1.45	0.78	0.88	0.04
P30101	PDIA3_HUMAN	PDIA3	Protein disulfide-isomerase A3	1.04	0.03	1.29	0.18
P08865	RSSA_HUMAN	RPSA	40S ribosomal protein SA	3.04	0.14	1.16	0.32
Q9Y230	RUVB2_HUMAN	RUVBL2	RuvB-like 2	0.59	0.70	1.00	0.07
P78371	TCPB_HUMAN	CCT2	T-complex protein 1 subunit beta	1.12	0.08	0.94	0.05
P38646	GRP75_HUMAN	HSPA9	Stress-70 protein, mitochondrial	0.67	0.55	0.44	0.16
P17987	TCPA_HUMAN	TCP1	T-complex protein 1 subunit alpha	0.29	0.67	0.20	0.33
P0DMV8	HS71A_HUMAN	HSPA1A	Heat shock 70 kDa protein 1A	0.20	0.07	0.68	0.55
P15311	EZRI_HUMAN	EZR	Ezrin	0.39	0.28	0.06	0.01
P15311	EZRI_HUMAN	EZR	Ezrin	1.64	0.37	0.49	0.23
P49368	TCPG_HUMAN	CCT3	T-complex protein 1 subunit gamma	0.33	0.86	0.09	0.30
P31948	STIP1_HUMAN	STIP1	Stress-induced-phosphoprotein 1	0.19	0.81	0.17	0.73
P49368	TCPG_HUMAN	CCT3	T-complex protein 1 subunit gamma	0.33	0.31	0.46	0.45
P12956	XRCC6_HUMAN	XRCC6	X-ray repair cross-complementing protein 6	0.77	0.37	0.75	0.45
P15311	EZRI_HUMAN	EZR	Ezrin	0.23	0.70	0.26	0.87
P06733	ENOA_HUMAN	ENO1	Alpha-enolase	1.0	0.64	0.2	0.04
P15311	EZRI_HUMAN	EZR	Ezrin	0.94	0.41	0.59	0.11
P27797	CALR_HUMAN	CALR	Calreticulin	0.70	0.08	0.66	0.05
Q9UQ80	PA2G4_HUMAN	PA2G4	Proliferation-associated protein 2G4	0.64	0.34	0.66	0.23
P28838	AMPL_HUMAN	LAP3	Cytosol aminpeptidase	0.65	0.27	0.52	0.16
P78371	TCBP_HUMAN	CCT2	T-complex protein 1 subunit beta	0.16	0.62	0.15	0.84
P68363	TBA1B_HUMAN	TUBA1B	Tubulin alpha-1B chain	0.28	0.99	0.06	0.04
P25705	ATPA_HUMAN	ATP5A1	ATP synthase subunit alpha, mitochondrial	0.18	0.41	0.29	0.61
P31930	QCR1_HUMAN	UQCRC1	Cytochrome b-c1 complex subunit 1, mitochondrial	0.67	0.80	0.58	0.99
Q9UQ80	PA2G4_HUMAN	PA2G4	Proliferation-associated protein 2G4	1.55	0.35	0.51	0.09
P06733	ENOA_HUMAN	ENO1	Alpha-enolase	1.10	0.58	1.12	0.46

P04083	ANXA_HUMAN	ANXA1	Annexin A1	0.43	0.04	0.45	0.03
P06733	ENOA_HUMAN	ENO1	Alpha-enolase	0.74	0.56	0.91	0.77
P07355	ANXA2_HUMAN	ANXA2	Annexin A2	0.31	0.30	0.30	0.28
P32322	P5CR1_HUMAN	PYCR1	Pyrroline-5-carboxylate reductase 1, mitochondrial			0.61	0.95
Q13011	ECH1_HUMAN	ECH1	Delta(3,5)-Delta(2,4)-dienoyl-CoA isomerase, mitochondrial	0.84	0.39	0.45	0.02
P30041	PRDX6_HUMAN	PRDX6	Peroxiredoxin-6	0.24	0.69	0.57	0.01
Q16576	RBBP7_HUMAN	RBBP7	Histone-binding protein RBBP7	0.55	0.38	0.25	0.15
P00441	SODC_HUMAN	SOD1	Superoxide dismutase [Cu-Zn]	0.60	0.29	0.85	0.00
P32119	PRDX2_HUMAN	PRDX2	Peroxideroxin-2	0.53	0.55	0.62	0.64
P60174	TPIS_HUMAN	TPI1	Triosephosphate isomerase	0.57	0.96	1.05	0.14
P52565	GDIR1_HUMAN	ARHGDIA	Rho GDP-dissociation inhibitor 1	0.42	0.61	0.38	0.56
P52565	GDIR1_HUMAN	ARHGDIA	Rho GDP-dissociation inhibitor 1	0.10	0.02	0.88	0.09
P08865	RSSA_HUMAN	RPSA	40S ribosomal protein SA	0.73	0.99	0.70	0.87
P06733	ENOA_HUMAN	ENO1	Alpha-enolase	0.5	0.56	0.4	0.96
P63104	1433Z_HUMAN	YWHAZ	14-3-3 protein zeta/delta	0.60	0.75	0.91	0.28
P43487	RANG_HUMAN	RANBP1	Ran-specific GTPase-activating protein	1.52	0.19	0.57	0.47
P04792	HSPB1_HUMAN	HSPB1	Heat shock protein beta-1	0.82	0.22	0.15	0.35
P18669 [a]	PGAM1_HUMAN	PGAM1	Phosphoglycerate mutase				
P35232 [a]	PHB_HUMAN	PHB	Prohibitin				
Q99497 [b]	Park7_HUMAN	PARK7	Protein deglycase DJ-1				
Q14240 [b]	IF4A2	EIF4A2	Eukaryotic initiation factor 4A-II				
P00367 [b]	DHE3_HUMAN	GLUD1	Glutamate dehydrogenase 1, mitochondrial				

[a] Only found in HCT116 cells stably overexpressing miR-143 or miR-145 2-DE protein spot patterns; [b] only found in HCT116 cells stably overexpressing miR-145 2-DE protein spot pattern. *p*-value for each fold was obtained using Student's *t*-test.
